# Evaluation of a glaucoma patient

**DOI:** 10.4103/0301-4738.73688

**Published:** 2011-01

**Authors:** Ravi Thomas, Klaus Loibl, Rajul Parikh

**Affiliations:** 1 Queensland Eye Institute, Brisbane, Queensland, Australia; 2 University of Queensland, Queensland, Australia; 3 Shreeji Eye Institute and Palak’s Glaucoma Care Centre, Mumbai, India

**Keywords:** Primary open angle glaucoma, primary angle closure glaucoma, comprehensive glaucoma evaluation, gonioscopy, optic disc

## Abstract

The diagnosis of glaucoma is usually made clinically and requires a comprehensive eye examination, including slit lamp, applanation tonometry, gonioscopy and dilated stereoscopic evaluation of the optic disc and retina. Automated perimetry is obtained if glaucoma is suspected. This establishes the presence of functional damage and provides a baseline for follow-up. Imaging techniques are not essential for the diagnosis but may have a role to play in the follow-up. We recommend a comprehensive eye examination for every clinic patient with the objective of detecting all potentially sight-threatening diseases, including glaucoma.

This article on the evaluation of primary adult glaucomas is specifically written for the general ophthalmologist working in the unequally developed, but modern, country that is 21^st^century India. In this scenario, the recommended strategy for prevention of blindness is case detection of the established disease. The detection and management of most ocular pathology, including glaucoma, is the purview of the general ophthalmologist.[1,2]

The patient usually does not present with a diagnosis of glaucoma for evaluation. Irrespective of the presenting (perhaps trivial) complaints, if we are serious about prevention of blindness, it is the responsibility of the examining ophthalmologist to rule out all potentially serious ocular pathology, including glaucoma, in all patients who seek care. The diagnosis of glaucoma at a treatable stage can be achieved by a clinical examination using basic instrumentation that should be available in every general ophthalmologist’s office.

Glaucoma is a chronic optic neuropathy with typical structural damage in the optic disc, usually accompanied by or leading to corresponding functional changes in the visual field.[3–5] It is important to remember that “raised” intraocular pressure (IOP) is a causal risk factor for glaucoma and the only one that can be treated, but it is neither sufficient nor necessary for the diagnosis.[3–5]

It is also important to bear in mind that glaucoma is usually asymptomatic till the late stages, at which time the prognosis is poor. The diagnosis of end-stage glaucoma is straightforward and can be made by a medical student trained in the use of the ophthalmoscope. It is however best to detect the disease at a stage where the diagnosis is easily possible, yet intervention can alter the course of the disease and change the prognosis. Diagnosis in the earliest stages (such as preperimetric glaucoma) is ideal but far more difficult than and not as critical as in established disease. As early diagnosis comes with implications of “labeling” and life-long treatment,[1,6] it is best confirmed by a specialist using the experience and tools at their disposal. The diagnostic importance of concepts such as pretest probability, sensitivity specificity and likelihood ratios of symptoms, signs and tests and how they can be used to confirm a diagnosis is dealt with elsewhere.[7,8]

## History

Primary open angle glaucoma (POAG) and primary angle closure disease (PACD) are usually asymptomatic. A history of frequent changes of reading glasses may be suspicious, but is not sensitive or specific enough to be used clinically. A family history of the disease increases the risk of glaucoma up to eight-fold and mandates a careful examination.[9–12] All family members of a patient with glaucoma must undergo a comprehensive eye examination. Myopes are at higher risk for POAG and hypermetropes are at higher risk for PACD.[13,14]

A directed history helps to rule out causes for the presence of glaucoma. These include steroid use (in any form), trauma, uveitis, sleep apnea, severe blood loss and intracranial disease. We must also enquire about the use of systemic medications that may impact glaucoma management. For example, a topical beta blocker may not add significantly to the IOP-lowering effect for someone already on a systemic beta blocker. As another example, topical alfa-2 agonist is contraindicated if the patient is on monoamine oxidase inhibitors.

## Examination

The comprehensive eye examination we describe below is recommended as a routine for all ophthalmic patients. The comprehensive eye examination helps detect not just glaucoma but other potentially blinding ocular pathology as well. Such a comprehensive eye examination comprises:

- Visual acuity and refraction,
- external examination and assessment of ocular motility,
- examination of the pupil with special attention to the presence of a relative afferent pupillary defect,
- slit-lamp biomicroscopy,
- IOP measurement,
- gonioscopy to examine the angle of the eye,
- dilated examination of the optic disc and retina and
- visual fields: If glaucoma is suspected, automated perimetry is performed to detect functional defects in the visual field.

While the clinical diagnosis of glaucoma is usually based on a combination of IOP, gonioscopy, optic disc and visual field examination, these steps should always be carried out as a part of the comprehensive eye examination and not in isolation.

The prevalence of glaucoma is high enough and the implications serious enough to suggest that all patients seen in an eye care professional’s clinic undergo the comprehensive eye examination as well as the “directed” investigations.

In some instances, a diagnosis may not be possible during the course of one visit. In suspects and in those with very early disease, it may be necessary to repeat the entire examination after a period of observation.

We now describe the essential components of a comprehensive eye examination.[15]

### External examination of the eye

This may detect signs such as a subtle hemangioma or dilated episcleral veins, which suggest a secondary cause. The presence of ciliary conjunctival congestion suggests sinister intraocular pathology, including acute angle closure.

### Ocular motility

Detection of amblyopia or sensory exotropia may change the management plan.

### Examination of the pupil

Glaucoma is usually an asymmetric disease, and demonstration of a relative afferent pupillary defect is an important diagnostic clue. It may be a prognostic factor as well. A dilated pupil may be a sign of angle closure.

### Slit-lamp examination

**Figure 1:**
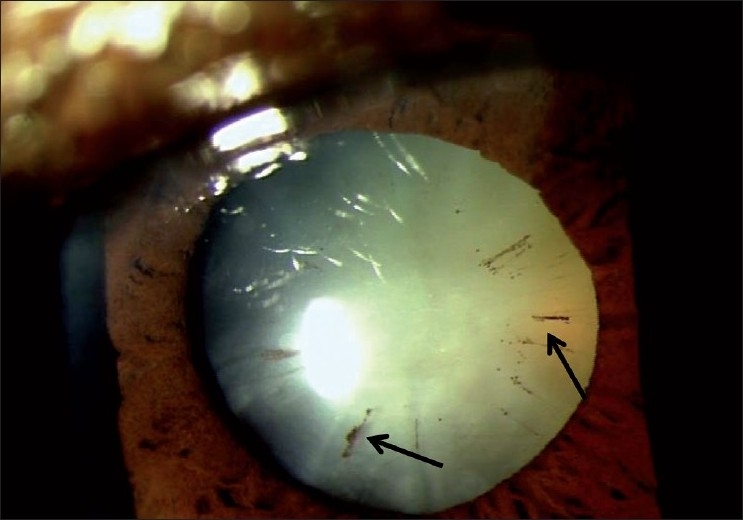
“Brown” stage of pseuduexfoliation (Arrow indicates “brown” stage of PXF)

This is performed both before and after dilatation and detects signs of pseudoexfoliation (PXE), pigment dispersion, uveitis or trauma. Pigment liberation following dilatation is highly suggestive of PXE and directs the search for subtle signs of this disease, like the early “brown” stage [Fig. 1]. Corneal edema detected during such an examination may underestimate IOP measurement. Posterior synechiae may explain distortion of the pupil. Findings in the corneal endothelium or iris may direct the search toward a secondary cause. Presence of “glaucamflacken” over the anterior lens surface indicates a previous acute attack of angle closure.

### Intraocular pressure

The IOP should be measured at every visit. The current gold standard is the Goldmann applanation tonometer attached to the slit lamp; the hand-held Perkins instrument can be used too. We are well into the 21^st^ century and the routine use of Schitoz tonometry is to be actively discouraged. It is important to remember that the Goldmann applanation tonometer, like any other measurement, may be subject to errors; measurements must be carefully performed to avoid erroneous readings. The measurement is also affected by corneal thickness. We suggest that the central corneal thickness (CCT) be measured in all ocular hypertensives and those suspected to have “normal tension” glaucoma, certainly before subjecting them to a neurological massage.[16] However, we must avoid the tendency to consider the CCT-corrected IOP to be “accurate.” And, there are other sources of error that cannot be accounted for by the CCT. Statistically, an IOP measured in the recommended manner, which, after correction for corneal thickness is raised beyond two standard deviations of the population mean, is suspicious. The two standard deviations value varies between populations, but >22 mmHg is a reasonable cut-off for the Indian population.[17–20] The tonopen (Reichert Ophthalmic Instruments, Depew, NY, USA), ICare Rebound Tonometer (Icare Finland Oy, Hevosenkenkä 3, Finland) and air puff tonometers have a place in busy clinics, but all abnormal values should be repeated and then confirmed by Goldmann applanation. The pascal dynamic Counter tonometer (Pascal Tonometer, Swiss Microtechnology AG, Port, Switzerland) can provide a closer estimate of the intracameral IOP, and seems to have the least variability.

As with any other measurement, especially in the absence of other signs of glaucoma, we should not rely on a single reading.[7,8] A measurement obtained after dilatation may increase the “yield.” The measurements should be repeated especially if the disc is suspicious, other signs of the disease are present or the patient is considered high risk, for example by virtue of a family history. This is necessary not just to detect raised IOP but also to obtain a baseline for treatment.

In the presence of disc and field changes, if the IOP is “normal” or “low,” multiple readings obtained during different times of the day (and even night) may be desirable. This should be considered before initiating any expensive or invasive investigations to explain the disc and field changes. The principle of multiple readings, preferably obtained at different times of the day, applies even after treatment is initiated. Obtaining diurnal curves are difficult; the recent use of the water drinking test to predict the peak IOP and IOP fluctuation requires further study.[21]

### Gonioscopy

POAG is a diagnosis of exclusion. The name itself indicates that the signs of glaucoma must be present with an open angle, in the absence of other causes. The demonstration of an open angle is especially important in regions of the world where PACD is common. While India has a higher burden of PACD, this also presents an excellent opportunity for prevention using a simple intervention.[22]

**Figure 2:**
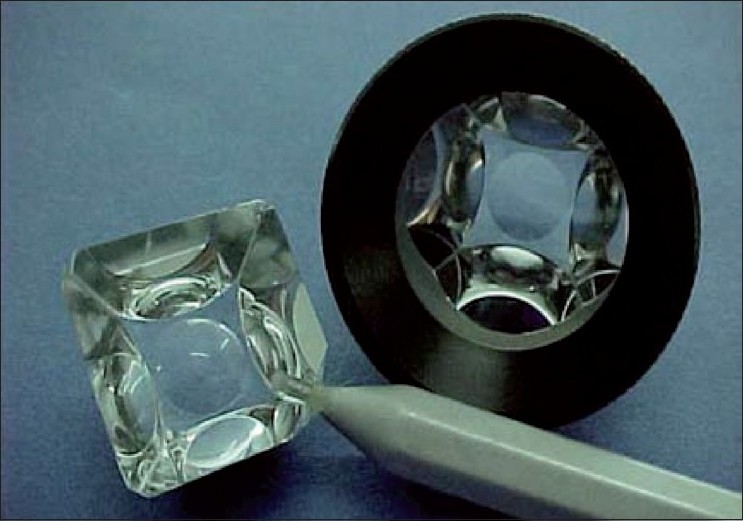
Indentation gonioscope

**Figure 3:**
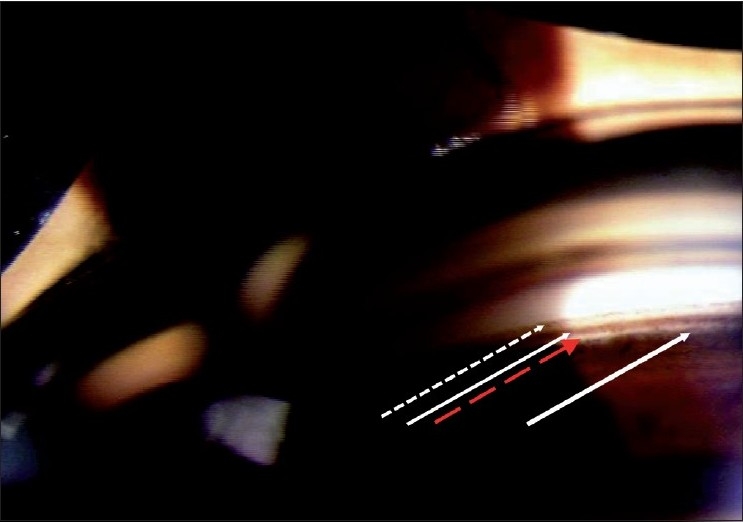
Normal angle anatomy (Broken arrow: Schwalbe’s line, White arrrow: Pigmented Trabecular Meshwork, Red arrow: Scleral Spur, Thick white arrow: Cilliary Body)

Gonioscopy is used to examine the angle of the anterior chamber, and is best performed using an “indentation” type of gonioscope [Fig. 2]. A four-mirror indentation gonioscope is the better choice; the lack of need for coupling fluid also makes the goal of routine gonioscopy easier. However, in the absence of a four-mirror gonioscope, using “manipulation” with a two-miror gonioscope to try and achieve indentation is an acceptable option. The features of an open angle are shown in Fig. 3.

**Figure 4:**
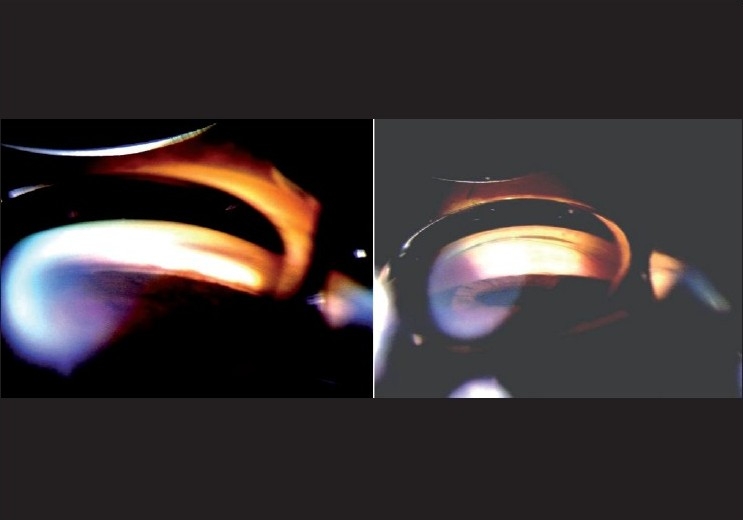
Gonioscopy showing angle with bright and dim illumination (Angle is open with bright illumination and the same angle is closed with appropriate testing conditons)

In determining if an angle is open or closed, the testing conditions are critical. If the examination is done in a bright room with a long slit beam that impinges on and constricts the pupil and/or with some pressure applied by the gonioscope, many angles will “open.” (Fig. 4 shows open and closed angle with bright and dim illumination.)

The ideal testing conditions include dim room illumination, minimal intensity of the slit-lamp illumination, a low slit-beam height such that light does not impinge on the pupil and no pressure on the eye with the gonioscope. Then, wait for 30–45 s for the pupil to dilate before deciding if the angle is open.

**Figure 5:**
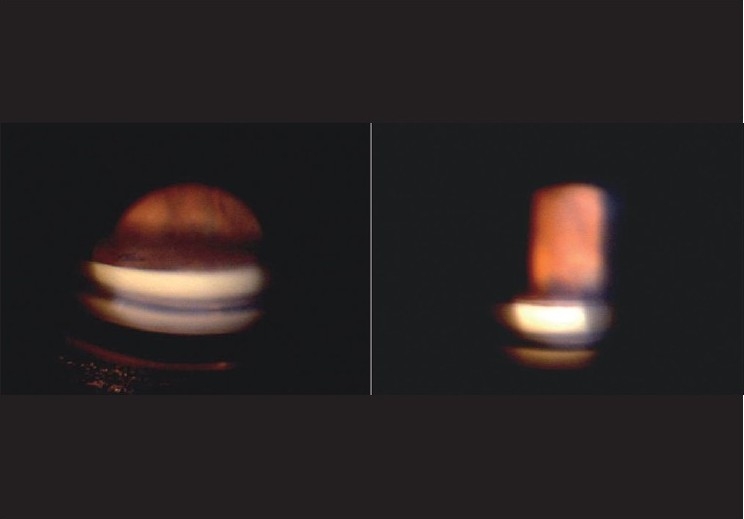
Gonioscopy showing “over the hill” angle (Gonioscopy showint no angle structure in straight ahead view. By tilting angle, “over the hill” view showing angle structures)

If, under these conditions, the posterior trabecular meshwork (PTM) is not seen, the patient is asked to look toward the mirror in order to obtain an “over the (iris) hill view” of the angle. If >180 degrees of the PTM is seen with such an “over the hill view” under the specified testing conditions, without any pressure on the eye, the angle is considered open [Fig. 5]. If not, the patient is considered a primary angle closure suspect.

**Figure 6:**
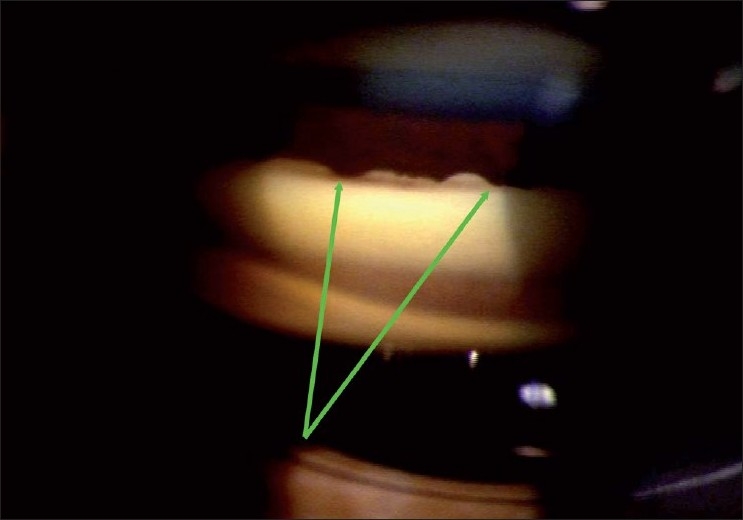
Gonioscopy showing peripheral anterior synechiae (Arrow showing peripheral anterior synechia)

The next step is to increase the illumination and the slit height to constrict the pupil and perform “indentation” with the goinoscope to look for other signs of pathology in the angle. These may include peripheral anterior synechiae [Fig. 6], a consequence of angle closure or inflammation, signs of PXE, trauma, old hemorrhage, inflammation or new vessels. The authors recently encountered another rare case where trabecular precipitates were the only evidence of inflammation in a patient being treated as POAG.[23] Investigations led to the diagnosis of uveitic glaucoma secondary to sarcoidosis.

If other signs are absent, and the angles are open under the conditions described above, then, in the presence of disc and/or field changes, we consider a diagnosis of POAG.

It is also important to remember that gonioscopy is not a one-time examination. A patient with POAG can develop an angle closure component over time. Gonioscopy should be repeated at least annually, if the signs of the disease change, and also after interventions like iridotomy or trabeculectomy.

### Role of the van Herrick tests and angle imaging

The van Herick test has been suggested as a screening test for angle closure. The sensitivity and specificity of this test are such that a negative test does not rule out angle closure and a positive test still requires a gonioscopy.[1,24] The presence of a positive van Herrick and a raised IOP is highly specific, and almost pathognomonic, of closure, but gonioscopy is still required for management.[1,24] Accordingly, if the philosophy is one of case detection and management, then the van Herrick test does not really help.

Angle imaging techniques such as the ultrasound bio-microsope and anterior segment optical coherence tomography (OCT) have not replaced gonioscopy and are not necessary for routine clinical use.

## Optic Disc and Nerve Fiber Layer (NFL) Examination

A magnified, preferably stereoscopic, examination of the optic disc using a 60-90 diopter (D) lens or a contact lens with the slit lamp is the ideal method of examining these structures.[15] Retinal examination also requires an indirect ophthalmoscope. The indirect ophthalmoscope alone is not good enough to comment on the optic disc. In experienced hands, the direct ophthalmoscope can provide valuable information too.

Stereo-photographs are the current gold standard but the optic disc findings should at least be documented, preferably with a drawing or imaging for comparison with future examinations.

**Figure 7:**
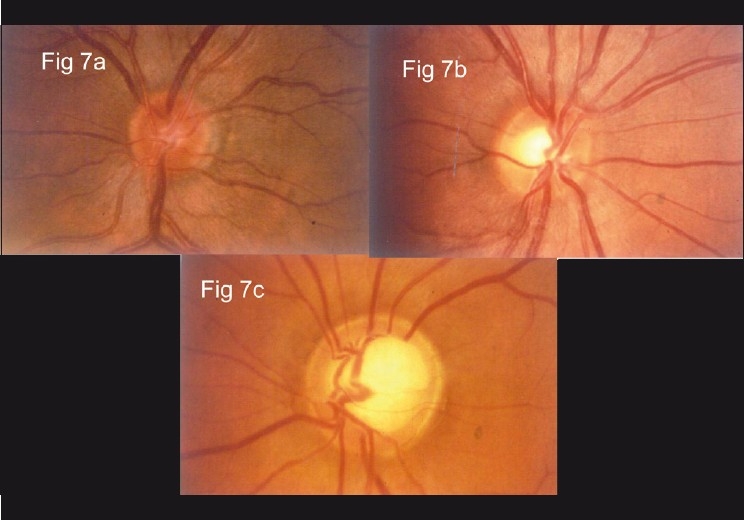
Relation between optic disc size and cup [(a) Small disc and has a small cup. (b) Medium sized disc with a larger cup. (c) Large disc and a large cup]

**Figure 8:**
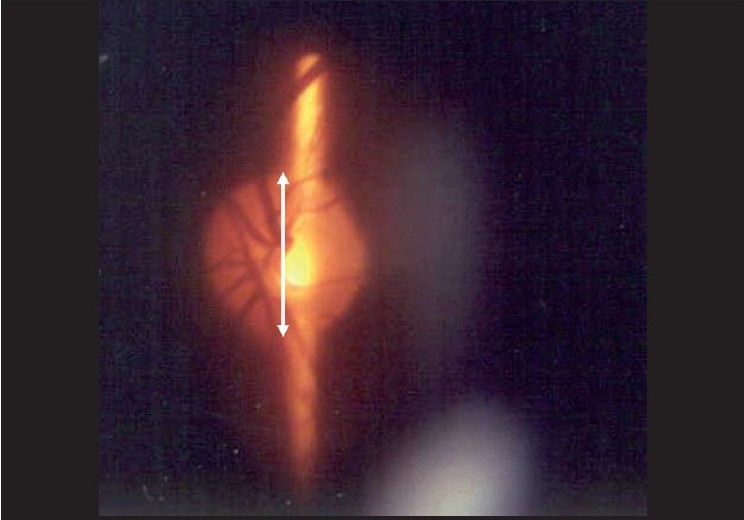
Estimation of optic disc size (Narrrow the vertical height of the slit beam to match the disc height)

The structural changes in the optic disc in glaucoma are numerous; the diagnosis is based on a combination of signs.[25] The most commonly used sign for the diagnosis of glaucomatous damage is an “increased” cup to disc ratio (CDR). Generally, an arbitrary statistical cut-off of 0.7:1 is considered to be suspicious. More so if the cup is vertically oriented. The CDR can be fallacious and should not be used in isolation. The reason is as follows: about a million plus axons exit the eye through the optic disc, forming the “neuro-retinal” rim of the optic disc. Think of the cup as the “space” that is left over after these axons have been “accommodated” in the disc. The size of the optic disc varies considerably; the “space left over,” that is the cup, has to vary with the size. Accordingly, a small-sized disc may not be entitled to any cup and a large-sized disc is entitled to a very large cup, beyond the 0.7:1 cut-off [Fig. 7]. The CDR can be useful, but only if it is related to the size of the disc. The size of the disc can be easily estimated on the slit lamp with a 60 D lens. The magnification factor for the 60 D lens is 1, for 78 D is 1.13 and for 90 D is 1.41. A narrow slit-beam height is adjusted vertically till it just encompasses the margins of the optic disc [Fig. 8].

A “normal”-sized disc in India has a vertical diameter of approximately 2.0 mm.[25] It is not important to obtain an actual measurement as it is to get a feel for whether a disc is small, medium or large. As with any other examination, this only becomes possible after examining and measuring a large number of discs. The question to ask is: “Is this disc physiologically allowed to have this sort of cup”? A small cup, like 0.3, usually considered to be in the normal range, may not be physiological in a small disc; on the other hand, a large cup may be physiological in a large disc. In other words, a small cup may be abnormal in a small disc and a large cup may be normal for a large disc.

CDR is also useful in two other situations. If, after accounting for a difference in size of the two discs, the CDR in the two eyes differs by more than >0.2, it is suspicious for glaucomatous damage. A loss of rim (increase in the cup) over time is pathognomonic of glaucoma.

It is important to remember that the cup and the CDR are only a surrogate for the tissue that we really want to examine, i.e. the part of the optic disc that is occupied by the axons: the neuroretinal rim (NRR). It is these changes in the NRR that suggest pathology.

Rather than the usual CDR, we prefer to document the rim to disc ratio in the superior, superotemporal, inferotemporal, inferior and nasal areas of the disc. A rim to disc ratio of under 0.1:1 should be considered pathology until proved otherwise. Rim to disc ratio also allows better monitoring.[26,27]

### Changes in the NRR

**Figure 9:**
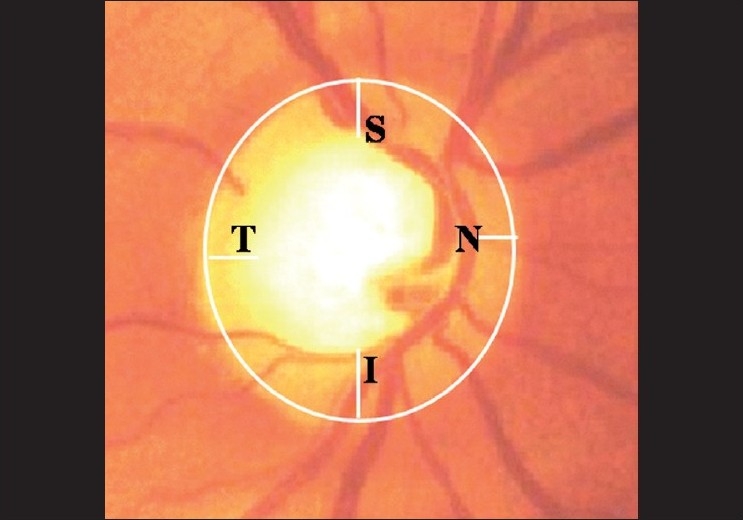
Neuro retinal rim: ISNT rule (Normally, inferior rim is thicker than superior rim, which in turn is thicker than nasal rim. Temporal rim is thinnest)

**Figure 10:**
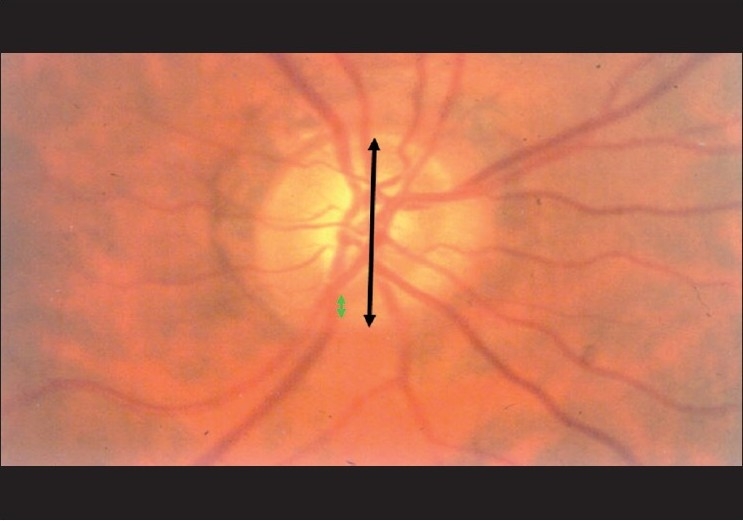
Optic disc showing early glaucoma (Loss of ISNT rule. Note that superior rim is thicker than inferior rim)

**Figure 11:**
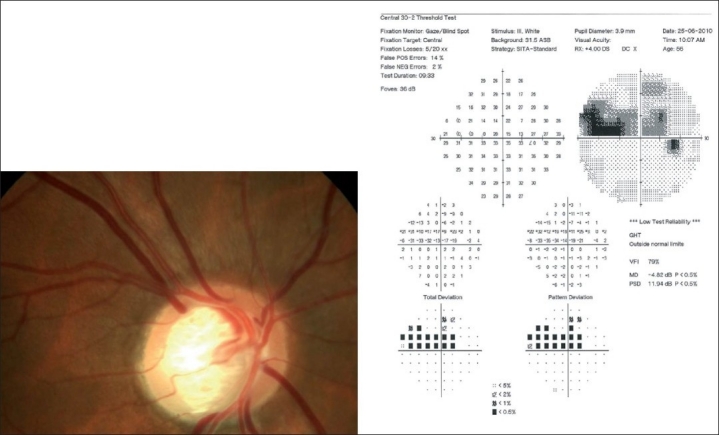
Optic disc with NOTCH

**Figure 12:**
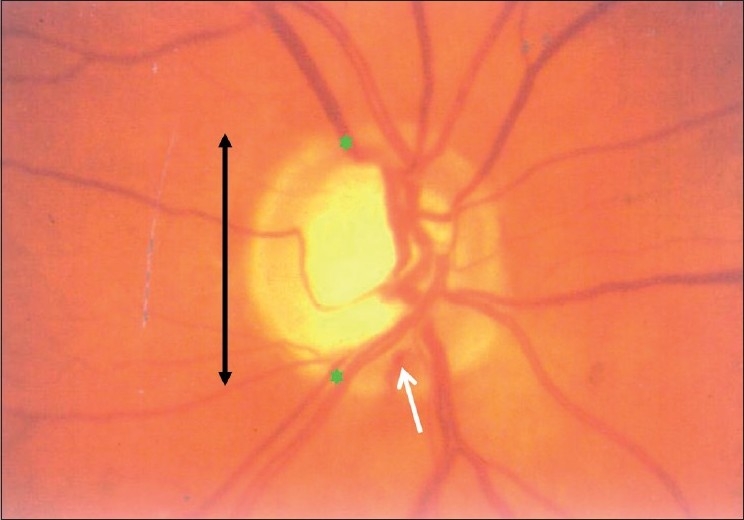
Optic disc showing disc hemorrhage (Rim to disc ratio <0.1:1 as seen here indicates glaucoma. Whith arrow indicates disc haemorrhage)

**Pattern:** The NRR is usually thickest inferiorly, followed by superior, nasal and temporal [Fig. 9]. This is the “ISNT” rule, seen in 80% of the nornals. While that is not specific enough, and there may be normal variations, any change in this pattern is suspicious[25] [Fig. 10]. If the inferior rim is thinner than the superior, that could suggest pathology. Certainly, an inferior or superior rim that is equal to or thinner than the temporal rim is highly suspicious. The temporal rim should be the thinnest. Localized narrowing of the inferior or superior rim that does not extend to the rim is also suspicious. If the rim extends to the edge of the disc for a clock hour it is called a notch. A notch is characteristic of glaucoma and usually produces a functional field defect too [Fig. 11]. A hemorrhage that touches the neuroretinal rim is specific but not sensitive for glaucoma [Fig. 12]. Pallor of the rim is not a sign of glaucoma. Pallor of the rim outside the area of loss or out of proportion to the “cupping” is suggestive of other neurological causes. Peripapillary choroidal atrophy is a soft sign of glaucomatous damage. It is significant if associated with other signs, or if it increases in size.

**Figure 13:**
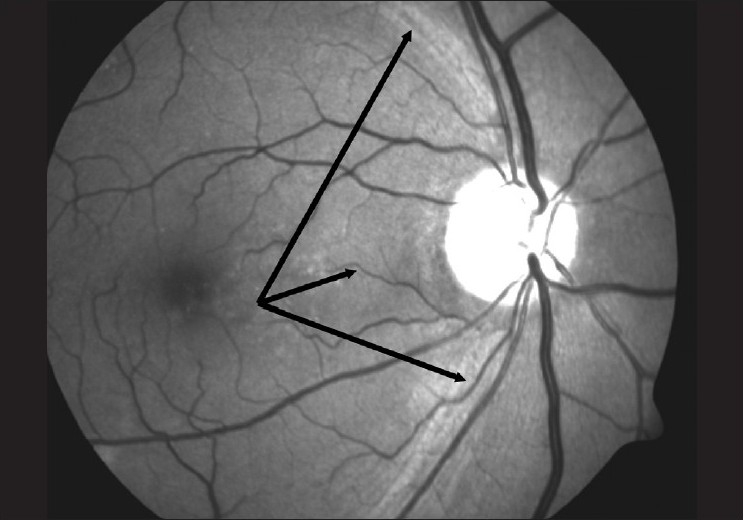
Red free optic disc photograph (Normal RNFL pattern: Bright dark bright pattern)

**Nerve Fiber Layer Defect (NFLD):** The gold standard for the examination of the NFL is red free photography, but the NFL can be examined clinically using the green filter on the slit lamp or ophthalmoscope. It is sometimes clearly seen on indirect ophthalmoscopy too, both with and without the green filter. The normal arcuate NFL is seen as fine bright striations. When viewed from the superior to the inferior arcuate area, the NFL has a bright, dark, bright pattern, the “bark” being the region between the disc and the macula [Fig. 13]. The inferior arcuate NFL has a larger area and is more clearly seen than the superior arcuate NFL, consistent with the NRR thickness.

**Figure 14:**
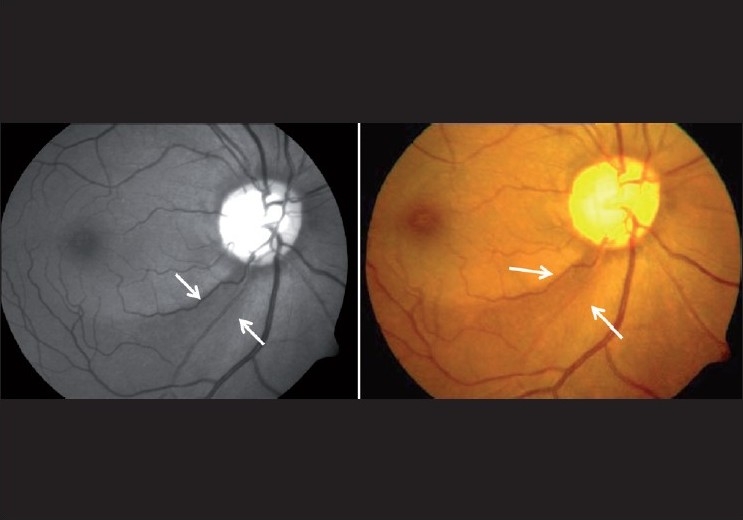
Optic photograph showing wedge-shaped defect (Arrow indiectes wedge shaped RNFL defects)

A localized NFLD appears as a dark wedge that follows the pattern of the NFL and increases in width toward the periphery [Fig. 14]. Such a defect must be wider than an arteriole, touch the edge of the disc and increase in width toward the periphery. Such defects have a strong predictive value for future functional changes. The specificity is very high but the sensitivity is poor.[28] The defects are a definite sign of pathology, but can occur in diseases other than glaucoma too.

**Figure 15:**
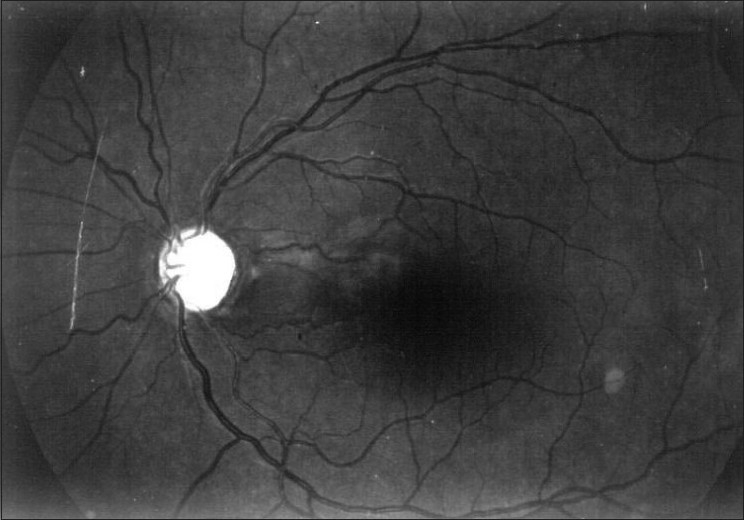
Red free photograph showing diffuse nerve fiber layer defects (Note that bright dark bright pattern is lost and it appears completely dark)

Diffuse NFLDs are more difficult to detect. The normal bright, dark, bright pattern is lost. The pattern looks more like dark, dark, dark [Fig. 15]. Better visibility of the superior NFL as compared with the inferior is also suspicious. The diagnosis of glaucomatous changes in the optic nerve is usually based on a combination of the above signs.[25–27] For example, in a disc with a notch as well as an NFLD, the combined specificity is high enough to “rule in” glaucoma. Similarly, in a disc with a thinning of the rim as well as an optic disc hemorrhage, the specificity is again high enough to rule in glaucoma. On the other hand, the sensitivity of individual signs is not high enough to rule out glaucoma unless most, or all, signs are absent.

Jost Jonas usually teaches three rules for optic disc examination in glaucoma diagnosis (Jonas JB, personal communication). Until proved otherwise:

- all glaucoma suspects have NFL defects,
- all glaucoma suspects have optic nerve hemorrhage and
- all myopes have glaucoma (myopes are at a higher risk for glaucoma).

As we are serious about detecting glaucoma at a stage when we can prevent visual disability, we have added the following rule:

- Unless proved otherwise, all optic discs have glaucomatous changes (and all angles are closed). The rule emphasizes that in order to detect glaucoma, we must have a high index of suspicion and examine all patients carefully. The optic disc should be examined at every visit. Depending on the course of the disease, documentation should be performed every 6–12 months.

### Imaging techniques for examining the optic disc

The optic nerve and/or nerve fiber layer imaging techniques include the Heidelberg Retinal Tomograph (HRT III), OCT and the NFL Analyzer (GDx VCC). The World Glaucoma Association consensus on imaging states that these instruments lack the sensitivity and specificity for routine clinical use. In the hands of experts, however, they may provide valuable clinical information.[29] These instruments can help corroborate our suspicions and help support our diagnosis, but are not required for routine clinical diagnosis. If we have to make a choice between automated perimetery or one of the imaging techniques for diagnosis, we would suggest the automated perimeter. We do feel that the imaging devices may have a potential for documenting and detecting change, and have a major role to play in this important area.

## Visual Field

Glaucoma is a potentially blinding disease because it causes defects in the visual field, which affect the visual function. Once such a defect is detected, diagnosis and management decisions become clearer. The detection of field defects and their progression (or stability) is therefore extremely important in glaucoma management. Like any other test, a visual field is obtained only if there is a suspicion of disease. A field “defect” in a person whose examination is normal is likely to be a false positive. If there is no suspicion of disease, do not obtain fields (or any other test, including imaging). The current gold standard for examination of the visual fields is a full-threshold automated perimetry. The examination of visual fields is a test with a very strong subjective element. Automated perimetry attempts to make this subjective test as objective as possible. In order to maintain this objectivity, we must use calibrated perimeters that have been previously validated.

**Figure 16:**
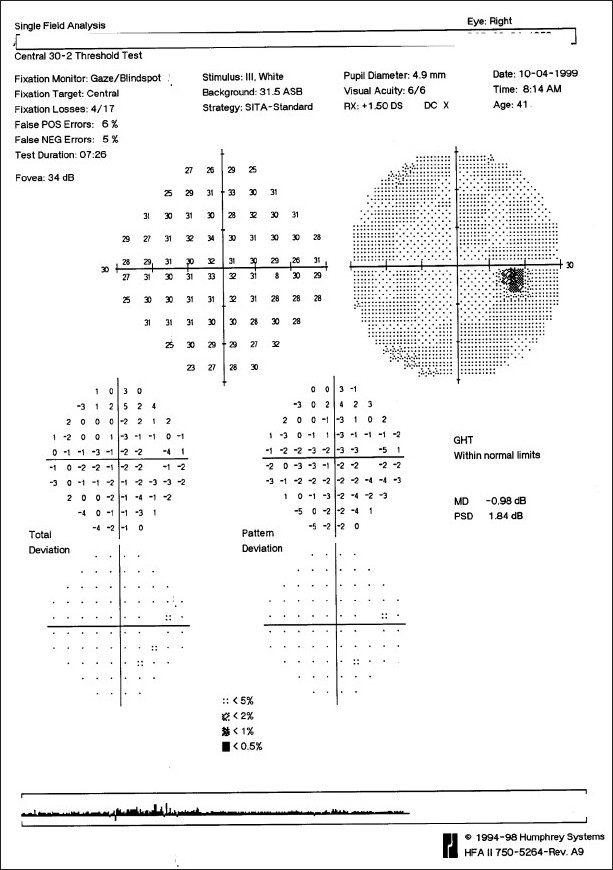
Normal visual field

**Figure 17:**
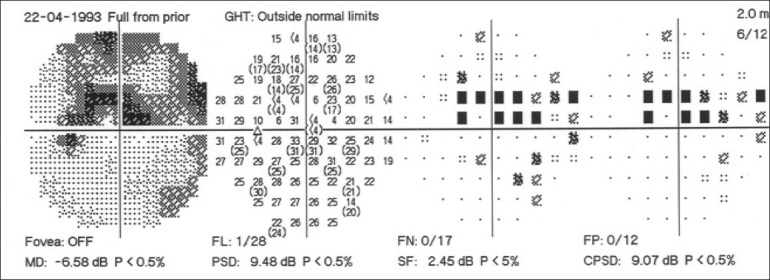
Visual field with an early glaucomatous defect

**Figure 18:**
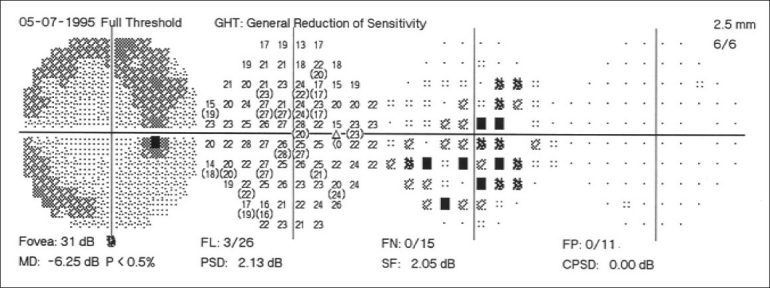
Visual field in patients with media opacity

Automated perimetry has a learning curve, and it is best not to rely on the first two fields. The perimetry printout is analyzed systematically in Zones.[30] A normal visual field is shown in Fig. 16. A field with an early glaucomatous defect is shown in Fig. 17. The field defects in glaucoma are usually localized. A localized defect will show up in both the total deviation and the pattern deviation plots. A generalized depression is more characteristic of anterior segment-related causes, like cataract affecting the visual field. In such cases, the defects are limited to the total deviation plot [Fig. 18].

### Warning

Visual fields must never be interpreted in isolation. The field should (usually) correlate with structural changes in the optic disc and NFL. If there is definite structural damage but the field is normal, repeat the visual field. If there is a visual field defect, but no correlating structural damage, examine the disc again. If there is still no correlating structural damage, re-examine the disc using a contact lens for the best stereoscopic view.

**Figure 19:**
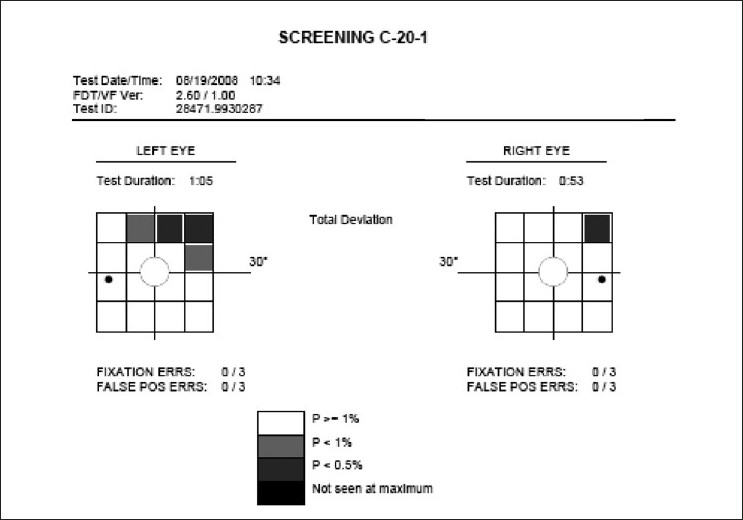
Glaucomatous visual field defect in the FDP 20 screening program (POAG patient with superior arcuate scotoma on WWP)

While the gold standard for perimetry is a full-threshold examination, in the presence of other signs of glaucoma, the presence of a repeatable defect in the 20-1 screening mode of a Frequency Doubling Perimeter (FDP) [Fig. 19] is sufficient evidence for a visual field defect due to glaucoma.[31,32] While it is not in the remit of this article, we feel that the demonstration of a repeatable functional defect is required before incisional surgery is considered. This can be done even with a Bjerrum’s screen, but an automated simple machine like the FDP is more likely to be used. The FDP is also capable of a full-threshold test, but has no follow-up capability.

The initial evaluation of a patient may or may not lead to a confirmed diagnosis or a decision to treat. Follow-up of suspects and patients at appropriate intervals for the detection of progression based on optic disc examination, imaging and serial visual fields is crucial to further decision making. It is therefore important to obtain baseline documentation of the optic disc and visual fields early in the course of the disease. Baseline fields should exclude the learning curve.

When do we suspect glaucoma? Family history of glaucoma, raised IOP (more than 22 mmHg), nonvisibility of the trabecular meshwork on gonioscopy, a “suspicious” optic disc (anything that looks out of the ordinary or outside the normal range), retinal nerve NFL, optic disc hemorrhage, high myopia, prevalence of glaucoma after the age of 60 years (which is high enough [5%] to suspect it on all persons above this age), long-term use of steroids and history of blunt trauma to the eye. If we want to (detect glaucoma early and) prevent blindness, all those who present to an eye care professional are glaucoma suspects and should at least undergo a comprehensive eye examination. This will not only detect glaucoma but also most potentially blinding conditions.

**Figure 20:**
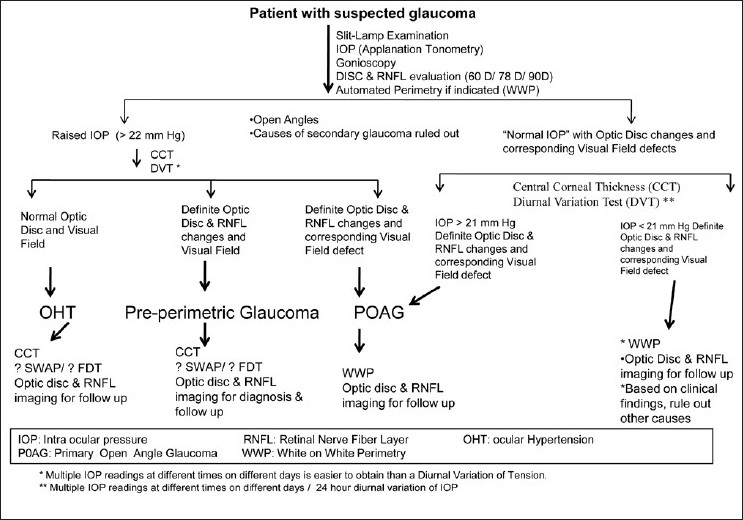
Evaluation of a glaucoma suspect (open angle glaucoma)

**Figure 21:**
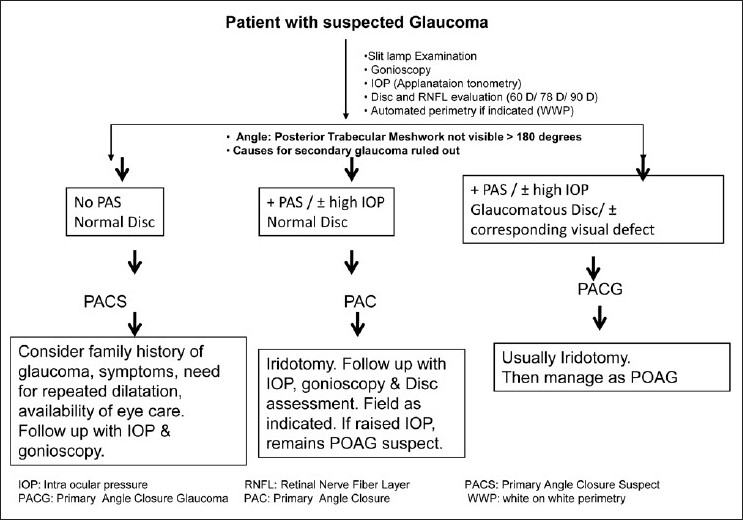
Evaluation of a glaucoma suspect (angle closure disease)

The work-up of a glaucoma suspect is shown in Figs. 20 and 21.

## Conclusion

The diagnosis of established glaucoma at a stage where treatment can prevent blindness involves the strategy of case detection. The general ophthalmologist should aim to detect all potentially serious ophthalmic pathology, including glaucoma. This requires a comprehensive eye examination, including slit lamp, IOP, gonioscopy and a dilated disc and retinal examination on all clinic patients. Automated perimetry should be obtained for all suspects. FDP is a cheap alternative to confirm the presence of glaucomatous visual field defects.
